# Bone turnover in elderly men: relationships to change in bone mineral density

**DOI:** 10.1186/1471-2474-8-13

**Published:** 2007-02-22

**Authors:** Tuan V Nguyen, Christian Meier, Jacqueline R Center, John A Eisman, Markus J Seibel

**Affiliations:** 1 Bone and Mineral Research Program, Garvan Institute of Medical Research, Sydney, Australia; 2 Faculty of Medicine, the University of New South Wales, Sydney, Australia; 3 Bone Research Program, ANZAC Research Institute, the University of Sydney, Sydney, Australia

## Abstract

**Background:**

It is not clear whether bone turnover markers can be used to make inference regarding changes in bone mineral density (BMD) in untreated healthy elderly men. The present study was designed to address three specific questions: (i) is there a relationship between bone turnover markers and femoral neck BMD within an individual; (ii) is there a relationship between baseline measurements of bone turnover markers and subsequent change in BMD; and (iii) is there a relationship between changes in bone turnover markers and changes in femoral neck BMD?

**Methods:**

The present study was part of the on-going Dubbo Osteoporosis Epidemiology Study, which was designed as a prospective investigation. Men who had had at least 3 sequential visits with serum samples available during follow-up were selected from the study population. Serum C-terminal telopeptide of type I collagen (sICTP), N-terminal propeptide of type I collagen (sPINP) and femoral neck BMD were measured by competitive radioimmunoassays. Femoral neck bone mineral density (BMD) was measured by a densitometer (GE Lunar Corp, Madison, WI). Various mixed-effects models were used to assess the association between the markers and changes in BMD.

**Results:**

One hundred and one men aged 70 ± 4.1 years (mean ± SD) met the criteria of selection for analysis. On average, sPINP decreased by 0.7% per year (p = 0.026), sICTP increased by 1.7% per year (p = 0.0002), and femoral neck BMD decreased by 0.4% per year (p < 0.01). Within-subject analysis indicated that changes in BMD were significantly associated with changes in sPINP (p = 0.022), but not with changes in sICTP (p = 0.84). However, neither baseline sPINP (p = 0.50) nor baseline sICTP (p = 0.63) was associated with subsequent changes in BMD. Moreover, changes in BMD were not significantly associated with previous changes in sPINP (p = 0.13) or sICTP (p = 0.95).

**Conclusion:**

These results suggest that in elderly men of Caucasian background, changes in sPINP were inversely related to changes in BMD within an individual. However, neither sPINP nor sICTP was sufficiently sensitive to predict the rate of change in BMD for a group of individuals or for an individual.

## Background

That the continuous remodeling process of bone formation and bone resorption can be measured by biochemical bone turnover markers is well-established; however, due to their substantial intra-subject variability, it remains controversial whether these indices are useful in the clinical setting. Being able to predict the change in bone mineral density (BMD) for an individual is of importance, as it may help to manage the disease progression and potentially provides an opportunity for intervention to reduce the fracture risk for the individual. A number of studies, mainly in women, have suggested that the association between bone turnover markers and change in BMD ranged from non-significant [1] or weak [2-4] to moderate association [5].

Bone loss (as measured by BMD) is less pronounced in men than in women [6,7], and the association between bone turnover markers and BMD is unclear. Cross-sectional studies in men have demonstrated that PINP, a bone formation marker, decreases with advancing age, but was paradoxically negatively correlated with BMD [8,9]. In a prospective study, again in elderly men, an increase in either PINP or ICTP was associated with increased loss of femoral neck BMD [10]. However, most of these studies were cross-sectional nature, and therefore, important questions on the within-subject relationships between bone turnover markers and change in BMD could not be addressed.

The present study was designed to examine the association between baseline measures and change in two bone markers and BMD in a sample of well-characterized elderly men. Three specific questions were asked: *First*, is there a relationship between bone turnover markers and femoral neck BMD within an individual? *Second*, is there a relationship between baseline measurements of bone turnover markers and change in BMD after controlling for unmeasured factors? These "unmeasured factors" were modeled by including a term for year, along with random factor terms for individual. *Third*, is there a causal relationship between changes in bone turnover markers and changes in femoral neck BMD?

## Methods

### Study subjects

The present study was part of the Dubbo Osteoporosis Epidemiology Study (DOES) for which the study design and protocol have been described elsewhere [11]. Briefly, DOES is a longitudinal, population-based study of risk factors for fracture and mortality. The study was approved by the St Vincent's Hospital Ethics Committee. Written informed consent was obtained from each subject. The study was initiated in mid-1989 and has been on-going since. By February 2003, 989 men and 1661 women had participated in the study.

Because the aim was to assess the association between changes in markers of bone turnover and changes in BMD, the present study included only men who have been longitudinally followed for at least 3 visits with serum samples available during follow-up period. Men with bone diseases, fractures, acute or chronic illnesses (i.e. cardiac failure, renal and liver insufficiency, and cancer), hypogonadism, or men on any medication affecting bone metabolism (i.e., glucocorticoids, calcitonin, anticonvulsants, and vitamin D or analogues) were excluded from the study. Ultimately, data from 101 men were included in the analysis. The median duration of follow-up was 6 years. All men have been scheduled for periodical visits, separated by an average of 2 years.

### Measurements

After obtaining written informed consent, subjects were interviewed by a nurse co-ordinator who administered a structured questionnaire to collect data including age, anthropometric variables, life-style and clinical. Serum samples were collected in the non-fasting state and stored at -80°C until analysis.

A marker of bone formation, serum N-terminal propeptide of type I collagen (sPINP) and a marker of bone resorption, serum C-terminal telopeptide of type I collagen (sICTP) were measured in duplicate by competitive radioimmunoassays (Orion Diagnostica, Espoo, Finland) [12,13]. The reproducibility of sPINP was 3.1 to 7.7%, with a male reference range being 21 to 78 μg/L. The reproducibility of sICTP was 6.7 to 8.5%, with a male reference range being 1.4 to 5.2 μg/L. All laboratory analyses were carried out in batches with all samples from a single subject run in one assay. The same batch of the respective assay was used for all measurements.

BMD was measured at the lumbar spine and femoral neck by dual energy X-ray absorptiometry using a LUNAR DPX densitometer (GE Lunar Corporation, Madison, WI, USA). The coefficient of reliability of BMD in our institution in normal subjects is 0.96, and the coefficient of variation was 1.5% at the proximal femur [14]. Quality control was performed regularly by using phantom to ensure the reliability of the densitometer. All BMD measurements were performed by the same operator and the same DXA instrument by a standardized protocol of measurement.

The primary dependent (outcome) variable used in this study was change in femoral neck BMD. This choice was based on the fact that in elderly men femoral neck BMD measurement is less likely to be affected by degenerative changes than lumbar spine BMD measurement. Thus, the use of femoral neck BMD as an outcome variable permitted a more reliable and valid way to examine the true relationship between PINP and ICTP and bone mineral density.

### Statistical analysis

To take full advantage of the hierarchical structure and longitudinal nature of the study, a mixed-effects linear model was considered. Various mixed-effects models containing both fixed and random effects and adjusted for the within-subject correlation [15] were considered. Model development was guided by the research questions. Details of these statistical models are shown in the Appendix. Briefly, the analysis began with the estimation of the baseline value and rate of change in BMD for each individual, assuming a linear function of time. The baseline value here refers to the intercept of an individual [15]. The collection of the rates of change across individuals was assumed to be normally distributed with a mean *β *and variance σβ2
 MathType@MTEF@5@5@+=feaafiart1ev1aaatCvAUfKttLearuWrP9MDH5MBPbIqV92AaeXatLxBI9gBaebbnrfifHhDYfgasaacH8akY=wiFfYdH8Gipec8Eeeu0xXdbba9frFj0=OqFfea0dXdd9vqai=hGuQ8kuc9pgc9s8qqaq=dirpe0xb9q8qiLsFr0=vr0=vr0dc8meaabaqaciaacaGaaeqabaqabeGadaaakeaaiiGacqWFdpWCdaqhaaWcbaGae8NSdigabaGaeGOmaidaaaaa@3131@. To address the first question (i.e., within-subjects correlation between bone turnover markers and BMD), a slight modification of the above model was considered in that association between bone turnover markers and BMD, the variation of BMD within an individual was modeled as a linear function of the individual's sPINP or sICTP over time. To address the second question (i.e., association between baseline bone turnover markers and subsequent changes in BMD), the individual intercepts and slopes of the outcome-time model were modeled as a linear function of baseline measurements of PINP and ICTP. The SAS PROC MIXED procedure [16] was used to derive estimates of the mixed-effects model parameters. Finally, to address the causative relationship between change in bone turnover markers and change in BMD, a first-order autoregressive model was used. In this model, the difference in BMD between visit 3 and visit 2 (*BMD*_3-2_) was modeled as a linear function of the difference in PINP (or ICTP) between visit 2 and visit 1 (*PINP*_2-1 _or *ICTP*_2-1_) such that *BMD*_3-2 _= *α *+ *β *(*PINP*_2-1_) + *γ *(*ICTP*_2-1_) + *e*, where *e *is the residual error term, and *α*, *β *and *γ *are regression coefficients to be estimated from the sample of 101 individuals. The estimates of these parameters were obtained by the least squares method with PROC REG of the SAS systems [17].

## Results

**Figure 1:**
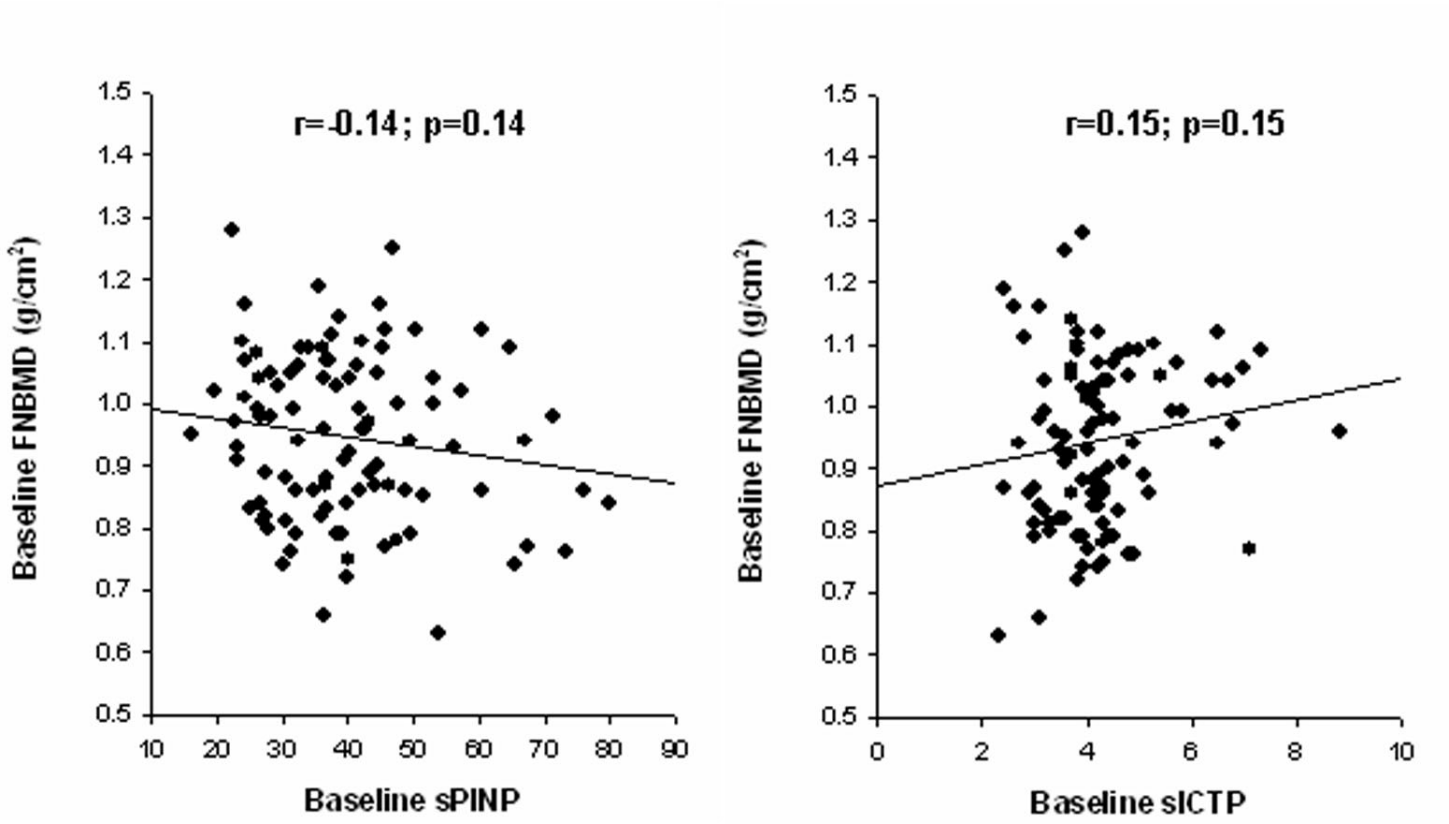
Relationship between baseline sPINP (left panel) or sICTP (right panel) and femoral neck BMD.

The 101 men had been followed for a median of 6 years (range: 4 to 10 years). At baseline (first visit) the men's average age was 70 years (SD: 4.1 years) (Table 1). All bone turnover markers and BMD measurements were normally distributed with no evidence of outliers or skewness of distribution. Cross-sectionally, there was a weak and statistically non-significant correlation between baseline femoral neck BMD and baseline PINP (*r *= -0.14; p = 0.14) and ICTP (*r *= 0.15, p = 0.14) (Figure 1).

### Rates of change in BMD, sPINP and sICTP

In longitudinal analyses (Table 2), femoral neck BMD decreased by 3.5 ± 1.0 mg/cm^2 ^per year (mean ± SE; p < 0.001), while lumbar spine BMD increased by 7 ± 1.3 mg/cm^2 ^per year (p < 0.001). When expressed in percentage terms, these changes were equivalent to -0.4% per year for the femoral neck and +0.6% per year for the lumbar spine. As indicated by the estimated variance of slopes (σβ2
 MathType@MTEF@5@5@+=feaafiart1ev1aaatCvAUfKttLearuWrP9MDH5MBPbIqV92AaeXatLxBI9gBaebbnrfifHhDYfgasaacH8akY=wiFfYdH8Gipec8Eeeu0xXdbba9frFj0=OqFfea0dXdd9vqai=hGuQ8kuc9pgc9s8qqaq=dirpe0xb9q8qiLsFr0=vr0=vr0dc8meaabaqaciaacaGaaeqabaqabeGadaaakeaaiiGacqWFdpWCdaqhaaWcbaGae8NSdigabaGaeGOmaidaaaaa@3131@), the rates of change in femoral neck BMD varied significantly among individuals (44 ± 16; p = 0.004); however, the rate of change in lumbar spine BMD did not differ significantly among individuals (p = 0.47). The non-significant covariance (σαβ2
 MathType@MTEF@5@5@+=feaafiart1ev1aaatCvAUfKttLearuWrP9MDH5MBPbIqV92AaeXatLxBI9gBaebbnrfifHhDYfgasaacH8akY=wiFfYdH8Gipec8Eeeu0xXdbba9frFj0=OqFfea0dXdd9vqai=hGuQ8kuc9pgc9s8qqaq=dirpe0xb9q8qiLsFr0=vr0=vr0dc8meaabaqaciaacaGaaeqabaqabeGadaaakeaaiiGacqWFdpWCdaqhaaWcbaGae8xSdeMae8NSdigabaGaeGOmaidaaaaa@32CB@) suggests that the baseline and rate of change in BMD were not significantly correlated.

The marker of bone formation, sPINP, decreased at the rate of -0.4 ± 0.18 μg/L or 0.7% per year (p = 0.026), whereas the resorption marker, sICTP, increased by 0.08 ± 0.02 μg/L or 1.7% per year (p = 0.0002). There was no statistically significant variation in the rates of change in sICTP or sPINP among individuals.

When comparing the unconditional linear change model with the unconditional mean model, 34% of the within-subject variance in BMD was attributable to time. However, time explained a modest proportion of within-subject variance in sPINP (10%) and sICTP (7%).

### Relationships between BMD and sPINP or sICTP within individuals

Results of the mixed-effects analysis suggests that after adjusting for the effect of age, each unit increase in sPINP was associated with a decrease of 9.2 ± 4.4 mg/cm^2 ^(p = 0.022) in femoral neck BMD. However, there was no significant association between changes in sICTP and changes in femoral neck BMD (p = 0.84) (Table 3). Compared with the unconditional linear change model in Table 2, the inclusion of sPINP or sICTP in the model accounted for a further 0.5% and 0.1% reduction in the within-subject variance in BMD, respectively.

### Relationships between baseline sPINP or sICTP and change in BMD

**Figure 2:**
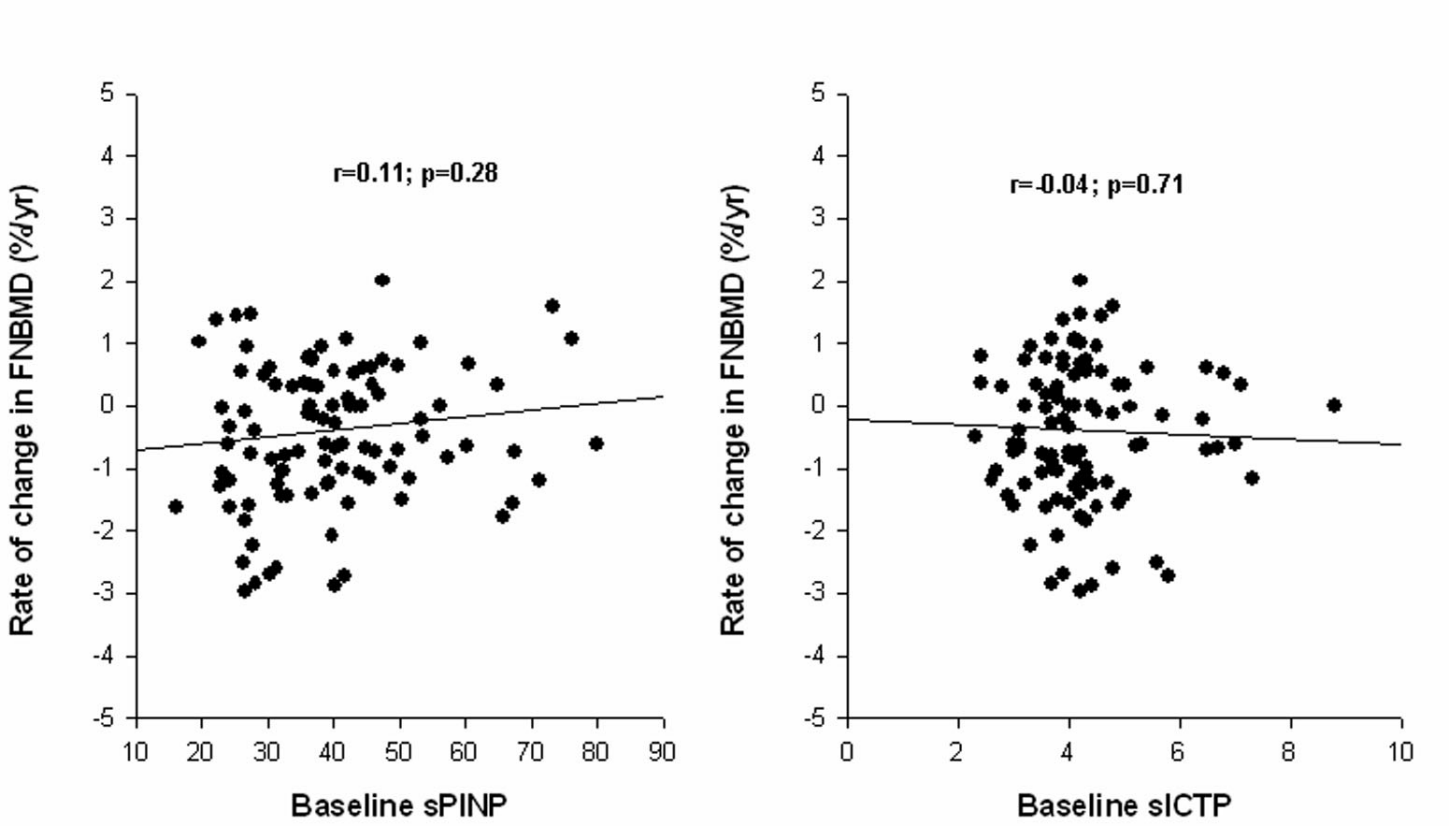
Relationship between change in femoral neck BMD and baseline sPINP (left panel) or sICTP (right panel).

Further analysis suggested that the rate of change in femoral neck BMD was not significantly correlated to baseline sPINP (p = 0.28) or sICTP (p = 0.71). Compared with the model shown in Table 3, the inclusion of baseline sPINP or sICTP did not significantly reduce the within-subject variance in BMD (Table 4 and Figure 2).

### Causal relationship between change in femoral neck BMD and change in sPINP

**Figure 3:**
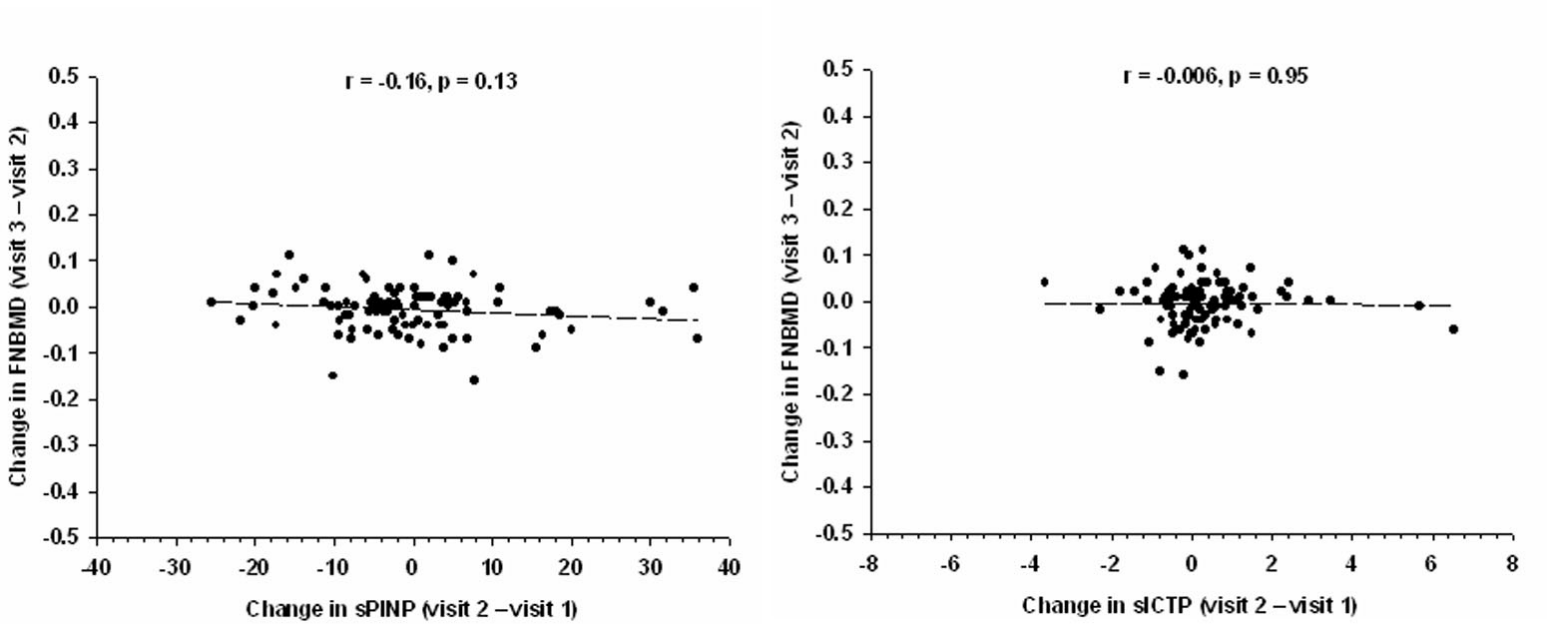
Relationship between change in femoral neck BMD (between visit 2 and visit 1) and change in sPINP (between visit 2 and visit 1) (left panel), and change in sICTP (right panel).

Changes in sPINP and sICTP between visit 2 and visit 1 were not significant predictors of subsequent change in femoral neck BMD between visit 3 and visit 2 (Figure 3). However, the relationship was consistent with results from the within-subject analysis. For instance, each unit increase in sPINP between visit 2 and visit 1 was related to a subsequent decrease in femoral neck BMD of 0.6 ± 0.4 mg/cm^2^, but the relationship was not statistically significant (p = 0.18).

## Discussion

Bone loss results from an imbalance between the two processes of bone resorption and bone formation, which can be measured by bone turnover markers. One of the suppositions in using bone turnover markers is to make inference regarding the magnitude of changes in bone density. In this study of untreated elderly men, a significant increase in bone resorption (as measured by sICTP), decrease in bone formation (as measured by sPINP), and decrease in femoral neck BMD were observed. Despite this, neither baseline measurement of, nor the change in, sPINP or sICTP predicted the subsequent rate of change in BMD.

It should be noted that the rate of change in BMD and the rate of change in either serum markers of bone turnover in this study was modest. However, such modest changes should not affect the predictive relationship between the bone turnover markers and BMD, should it exist, as the variance of rates of change was substantial in both markers and BMD. Furthermore, the current sample size had a more than 80% chance to detect a correlation of 0.2 at the significance level of 5% should the association exist: the sample size required to detect a correlation of 0.25 at the significance level of 5% is approximately 62 subjects). Therefore, the present study's non-significant results are unlikely to be due to the study's power or the distribution of bone loss of change in sPINP or sICTP.

In elderly women, a combination of three biochemical markers accounted for more than 50% of the variance in bone mineral density [18]. Skeletal turnover rates appear to be as much as 85% higher in elderly women with low bone mass, compared with women who have normal bone density. Furthermore, in a large prospective study of elderly postmenopausal women in Europe [19], levels of osteocalcin, *C*-telopeptides, and bone-specific alkaline phosphatase were all significantly greater in older than younger women. Furthermore, these bone formation markers were inversely related to femoral bone mineral density.

However, the situation in men remains largely unclear. In a relatively small longitudinal study of 24 men, whose BMD, sPINP and sICTP were measured 3 years apart, change in BMD was significantly and inversely correlated with change in sPINP (*r *= -0.24; p = 0.05) and change in sICTP (*r *= -0.24; p = 0.01) [10]. However, it was not clear from that study whether both factors were independent predictors of BMD change if they were considered simultaneously in the same model, or whether baseline sPINP and sICTP was predictive of subsequent change in BMD. These results together with those of the present study clearly suggest that the contribution of bone turnover markers to the prediction of change in BMD is minimal. Indeed, variation in sPINP and sICTP accounts for less than 1% of the variance of rates of change in BMD within subjects.

In interpreting the association between bone turnover markers and BMD, it is important to make distinction between a between-subject correlation from within-subject correlation between change in sPINP (or sICTP) and change in BMD. Previous studies [20,21] examined the between-subject correlation, whereas the present study primarily examined the within-subjects association. For example, it was observed in this study that although the between-subject correlation between sICTP and femoral neck BMD was statistically significant (*r *= 0.15, p = 0.03), the within-subject correlation was not. Thus, while individuals with higher/lower levels of sICTP may have lower/higher BMD, the increase in sICTP within an individual does not necessarily translating into a decrease in BMD. More importantly, there was no causal relationship between change in either sICTP or sPINP and change in BMD, as the preceding change in the markers was not a significant predictor of subsequent change in BMD.

A number of studies in women have suggested that a baseline measurement of bone resorption markers such as urinary CTx [19], urinary NTx [19], urinary free dPyr [22] and sICTP [23] was each associated with fracture risk [5]. Thus, although sICTP was not a significant predictor of change in BMD it is a predictive of fracture risk, independent of BMD. Indeed, in this cohort, baseline measurement of sICTP, but not sPINP, was found to be an independent predictor of fracture risk, such that a combination of sICTP and femoral neck BMD yielded a better predictive value than either measurement [24].

Measurements of bone turnover markers in serum are generally considered to be more reliable and more accurate than those measured in urine, as the confounding errors associated with creatinine measurement are largely eliminated. Nevertheless, the within-subject biological variation in sPINP and sICTP was around 20% [5], which in turn can be attributed to factors such as non-uniform rates of bone turnover, variable rates of metabolism and clearance, diurnal and seasonal variability, and other factors affecting pre-analytic variability. This relatively large variation in bone turnover markers can compromise the relationship between change in these markers and change in BMD within an individual.

Inaccuracy and unreliability of measurements, including within-subject random variability, can affect the delineation of the association between bone turnover markers and BMD or fracture risk in some epidemiological studies. Theoretically, if the coefficients of reliability of BMD and sPINP are *r*_1 _and *r*_2_, respectively, then it can be shown that the maximum squared correlation between the two variables is *r*_1_*r*_2 _[25]. In our study, *r*_1 _= 0.96 and *r*_2 _= 0.91, then the maximally expected squared correlation between BMD and sPINP is 0.87. However, in reality the correlation was 0.0196. Given the imprecision, this correlation could be about 0.0196/0.87 = 0.022. Therefore, it seems the imprecision of measurement did not account for the modest association that we observed in this study.

The decrease in sPINP and increase in sICTP observed in this study are indications of reduced bone formation and increased bone resorption activities. This is consistent with the established fact that androgen levels (free or available testosterone) decrease with advancing age [26,27]. Reduced testosterone is associated with bone loss in men [28], probably associated with a reduction in vitamin D levels leading to calcium malabsorption and reduced bone formation [29]. Recently, the important role of estrogen in the regulation of bone metabolism in men has been recognized [30], because estrogen controls bone resorption in elderly men [31]. The present study did not measure estrogen in these men, therefore it was not possible to examine the relationship between estrogen and the bone turnover markers. Although vitamin D was not measured in these men, in a study of men in the same cohorts from whom the present study's men were selected, the prevalence of vitamin D deficiency was extremely low (less than 1%). Thus, the present study's result was unlikely to be compromised by vitamin D status.

## Conclusion

In summary, the present study suggests that in a group of non-treated elderly men of Caucasian background, the change in the rate of bone formation, as measured by sPINP, was associated with the change in BMD within a subject. However, neither sPINP nor sICTP was sufficiently sensitive to predict the rate of change in BMD for a group of individuals or for an individual.

## Competing interests

The author(s) declare that they have no competing interests.

## Authors' contributions

TVN, CM, JAE and MJS were involved in the conceptual discussion and design of the study. CM carried out the biochemical analyses. TVN performed the statistical analysis and drafted the manuscript. JRC was involved in the data collection. TVN, JAE and JRC obtained the funding for the Dubbo Osteoporosis Epidemiology Study. All authors read and approved the final manuscript.

## Appendix: mixed-effects models

In what follows *i *denotes study subjects (*i *= 1, 2, 3, ..., 101), and *t *denotes visits (*t = *1, 2, 3). The measurement of each outcome variable, BMD, PINP, and ICTP, taken at the *t*th visit for the *i*th subject is denoted by *BMD*_*it*_, *PINP*_*it*_, and *ICTP*_*it*_, respectively. The duration of follow-up for the *t*th visit, calculated from the initial visit, is denoted by *T*_*it*_, where *T*_*i*1 _= 0. The change in each outcome variable for each subject was first modeled as a linear function of time (also called the unconditional linear growth model) as follows:

*BMD*_*it *_= *α*_*i *_+ *β*_*i*_*T*_*it *_+ *e*_*it *_    (1)

where *α *_*i *_denotes the BMD measurement at the initial visit (baseline value) and *β *_*i *_denotes the rate of change in BMD per year for the *i*th subject, and *e*_*it *_denotes the within-subject random variation. The baseline BMD of the *i*th subject was then modeled as a function of population mean, *α*, and a deviation from the population mean, *r*_*i*_: *α*_*i *_= *α *+ *r*_*i*_. Similarly, the rate of change for the *i*th subject was assumed to be equal to an overall mean rate of change, *β*, and a deviation from the mean associated with the *i*th individual, *s*_*i*_: *β*_*i *_= *β *+ *s*_*i*_. Therefore, the BMD values of the *i*th subject in (1) can now be written as:

*BMD*_*it *_= *α *+ *βT*_*it *_+ r_*i *_+ *s*_*i*_*T*_*it *_+ *e*_*it *_    (2)

*PINP*_*it *_and *ICTP*_*it *_were also modeled similarly. It is assumed that *e*_*it *_are independently normally distributed with mean 0 and variance σe2
 MathType@MTEF@5@5@+=feaafiart1ev1aaatCvAUfKttLearuWrP9MDH5MBPbIqV92AaeXatLxBI9gBaebbnrfifHhDYfgasaacH8akY=wiFfYdH8Gipec8Eeeu0xXdbba9frFj0=OqFfea0dXdd9vqai=hGuQ8kuc9pgc9s8qqaq=dirpe0xb9q8qiLsFr0=vr0=vr0dc8meaabaqaciaacaGaaeqabaqabeGadaaakeaaiiGacqWFdpWCdaqhaaWcbaGaemyzaugabaGaeGOmaidaaaaa@30E8@. The distributions of *r*_*i *_and *s*_*i *_are assumed to be normal with mean 0, variances of σα2
 MathType@MTEF@5@5@+=feaafiart1ev1aaatCvAUfKttLearuWrP9MDH5MBPbIqV92AaeXatLxBI9gBaebbnrfifHhDYfgasaacH8akY=wiFfYdH8Gipec8Eeeu0xXdbba9frFj0=OqFfea0dXdd9vqai=hGuQ8kuc9pgc9s8qqaq=dirpe0xb9q8qiLsFr0=vr0=vr0dc8meaabaqaciaacaGaaeqabaqabeGadaaakeaaiiGacqWFdpWCdaqhaaWcbaGae8xSdegabaGaeGOmaidaaaaa@312F@ and σβ2
 MathType@MTEF@5@5@+=feaafiart1ev1aaatCvAUfKttLearuWrP9MDH5MBPbIqV92AaeXatLxBI9gBaebbnrfifHhDYfgasaacH8akY=wiFfYdH8Gipec8Eeeu0xXdbba9frFj0=OqFfea0dXdd9vqai=hGuQ8kuc9pgc9s8qqaq=dirpe0xb9q8qiLsFr0=vr0=vr0dc8meaabaqaciaacaGaaeqabaqabeGadaaakeaaiiGacqWFdpWCdaqhaaWcbaGae8NSdigabaGaeGOmaidaaaaa@3131@, respectively, and a covariance of σαβ2
 MathType@MTEF@5@5@+=feaafiart1ev1aaatCvAUfKttLearuWrP9MDH5MBPbIqV92AaeXatLxBI9gBaebbnrfifHhDYfgasaacH8akY=wiFfYdH8Gipec8Eeeu0xXdbba9frFj0=OqFfea0dXdd9vqai=hGuQ8kuc9pgc9s8qqaq=dirpe0xb9q8qiLsFr0=vr0=vr0dc8meaabaqaciaacaGaaeqabaqabeGadaaakeaaiiGacqWFdpWCdaqhaaWcbaGae8xSdeMae8NSdigabaGaeGOmaidaaaaa@32CB@.

For interpretation, *α *represents the population mean of an outcome measure (i.e., BMD, PINP or ICTP), *β *represents the average linear annual rate of change in the outcome measure; σe2
 MathType@MTEF@5@5@+=feaafiart1ev1aaatCvAUfKttLearuWrP9MDH5MBPbIqV92AaeXatLxBI9gBaebbnrfifHhDYfgasaacH8akY=wiFfYdH8Gipec8Eeeu0xXdbba9frFj0=OqFfea0dXdd9vqai=hGuQ8kuc9pgc9s8qqaq=dirpe0xb9q8qiLsFr0=vr0=vr0dc8meaabaqaciaacaGaaeqabaqabeGadaaakeaaiiGacqWFdpWCdaqhaaWcbaGaemyzaugabaGaeGOmaidaaaaa@30E8@ represents the within-subject variance or the pooled deviation of each subject's measure around his "true" average; σα2
 MathType@MTEF@5@5@+=feaafiart1ev1aaatCvAUfKttLearuWrP9MDH5MBPbIqV92AaeXatLxBI9gBaebbnrfifHhDYfgasaacH8akY=wiFfYdH8Gipec8Eeeu0xXdbba9frFj0=OqFfea0dXdd9vqai=hGuQ8kuc9pgc9s8qqaq=dirpe0xb9q8qiLsFr0=vr0=vr0dc8meaabaqaciaacaGaaeqabaqabeGadaaakeaaiiGacqWFdpWCdaqhaaWcbaGae8xSdegabaGaeGOmaidaaaaa@312F@ is the between-subject variance of the measure; σβ2
 MathType@MTEF@5@5@+=feaafiart1ev1aaatCvAUfKttLearuWrP9MDH5MBPbIqV92AaeXatLxBI9gBaebbnrfifHhDYfgasaacH8akY=wiFfYdH8Gipec8Eeeu0xXdbba9frFj0=OqFfea0dXdd9vqai=hGuQ8kuc9pgc9s8qqaq=dirpe0xb9q8qiLsFr0=vr0=vr0dc8meaabaqaciaacaGaaeqabaqabeGadaaakeaaiiGacqWFdpWCdaqhaaWcbaGae8NSdigabaGaeGOmaidaaaaa@3131@ is the between-subject variance of the linear rates of change in the measure; and σαβ2
 MathType@MTEF@5@5@+=feaafiart1ev1aaatCvAUfKttLearuWrP9MDH5MBPbIqV92AaeXatLxBI9gBaebbnrfifHhDYfgasaacH8akY=wiFfYdH8Gipec8Eeeu0xXdbba9frFj0=OqFfea0dXdd9vqai=hGuQ8kuc9pgc9s8qqaq=dirpe0xb9q8qiLsFr0=vr0=vr0dc8meaabaqaciaacaGaaeqabaqabeGadaaakeaaiiGacqWFdpWCdaqhaaWcbaGae8xSdeMae8NSdigabaGaeGOmaidaaaaa@32CB@ represents the correlation between the true average and the rate of change. The estimates of *α*, *β*, σα2
 MathType@MTEF@5@5@+=feaafiart1ev1aaatCvAUfKttLearuWrP9MDH5MBPbIqV92AaeXatLxBI9gBaebbnrfifHhDYfgasaacH8akY=wiFfYdH8Gipec8Eeeu0xXdbba9frFj0=OqFfea0dXdd9vqai=hGuQ8kuc9pgc9s8qqaq=dirpe0xb9q8qiLsFr0=vr0=vr0dc8meaabaqaciaacaGaaeqabaqabeGadaaakeaaiiGacqWFdpWCdaqhaaWcbaGae8xSdegabaGaeGOmaidaaaaa@312F@, σβ2
 MathType@MTEF@5@5@+=feaafiart1ev1aaatCvAUfKttLearuWrP9MDH5MBPbIqV92AaeXatLxBI9gBaebbnrfifHhDYfgasaacH8akY=wiFfYdH8Gipec8Eeeu0xXdbba9frFj0=OqFfea0dXdd9vqai=hGuQ8kuc9pgc9s8qqaq=dirpe0xb9q8qiLsFr0=vr0=vr0dc8meaabaqaciaacaGaaeqabaqabeGadaaakeaaiiGacqWFdpWCdaqhaaWcbaGae8NSdigabaGaeGOmaidaaaaa@3131@, σαβ2
 MathType@MTEF@5@5@+=feaafiart1ev1aaatCvAUfKttLearuWrP9MDH5MBPbIqV92AaeXatLxBI9gBaebbnrfifHhDYfgasaacH8akY=wiFfYdH8Gipec8Eeeu0xXdbba9frFj0=OqFfea0dXdd9vqai=hGuQ8kuc9pgc9s8qqaq=dirpe0xb9q8qiLsFr0=vr0=vr0dc8meaabaqaciaacaGaaeqabaqabeGadaaakeaaiiGacqWFdpWCdaqhaaWcbaGae8xSdeMae8NSdigabaGaeGOmaidaaaaa@32CB@, and σe2
 MathType@MTEF@5@5@+=feaafiart1ev1aaatCvAUfKttLearuWrP9MDH5MBPbIqV92AaeXatLxBI9gBaebbnrfifHhDYfgasaacH8akY=wiFfYdH8Gipec8Eeeu0xXdbba9frFj0=OqFfea0dXdd9vqai=hGuQ8kuc9pgc9s8qqaq=dirpe0xb9q8qiLsFr0=vr0=vr0dc8meaabaqaciaacaGaaeqabaqabeGadaaakeaaiiGacqWFdpWCdaqhaaWcbaGaemyzaugabaGaeGOmaidaaaaa@30E8@ are shown in Table 2.

To address the first aim (e.g., is there an association between PINP or ICTP and BMD within individuals), model (1) was extended to accommodate the effect of PINP or ICTP:

*BMD*_*it *_= *α*_*i *_+ *β*_*i*_*T*_*it *_+ *γ*(*PINP*_*it*_) + *e*_*it*_

or *BMD*_*it *_= *α*_*i *_+ *β*_*i*_*T*_*it *_+ *γ*(*ICTP*_*it*_) + *e*_*it *_    (3)

where *α*_*i *_= *α *+ *r*_*i*_, *β*_*i *_= *β *+ *s*_*i *_as above, and *γ *_*i *_are assumed to be a fixed effect. Note that unlike *α *_*i *_and *β *_*i*_, the coefficient associated with PINP or ICTP is assumed to be a constant across subjects. Therefore, the BMD values of the *i*th subject can now be written as: *BMD*_*it *_= *α *+ *βT*_*it *_+ *γPINP*_*it *_+ *r*_*i *_+ *s*_*i*_*T*_*it *_+ *e*_*it *_or *BMD*_*it *_= *α *+ *βT*_*it *_+ *γICTP*_*it *_+ *r*_*i *_+ *s*_*i*_*T*_*it *_+ *e*_*it*_. The estimates of *α*, *β*, *γ*, σα2
 MathType@MTEF@5@5@+=feaafiart1ev1aaatCvAUfKttLearuWrP9MDH5MBPbIqV92AaeXatLxBI9gBaebbnrfifHhDYfgasaacH8akY=wiFfYdH8Gipec8Eeeu0xXdbba9frFj0=OqFfea0dXdd9vqai=hGuQ8kuc9pgc9s8qqaq=dirpe0xb9q8qiLsFr0=vr0=vr0dc8meaabaqaciaacaGaaeqabaqabeGadaaakeaaiiGacqWFdpWCdaqhaaWcbaGae8xSdegabaGaeGOmaidaaaaa@312F@, σβ2
 MathType@MTEF@5@5@+=feaafiart1ev1aaatCvAUfKttLearuWrP9MDH5MBPbIqV92AaeXatLxBI9gBaebbnrfifHhDYfgasaacH8akY=wiFfYdH8Gipec8Eeeu0xXdbba9frFj0=OqFfea0dXdd9vqai=hGuQ8kuc9pgc9s8qqaq=dirpe0xb9q8qiLsFr0=vr0=vr0dc8meaabaqaciaacaGaaeqabaqabeGadaaakeaaiiGacqWFdpWCdaqhaaWcbaGae8NSdigabaGaeGOmaidaaaaa@3131@, σαβ2
 MathType@MTEF@5@5@+=feaafiart1ev1aaatCvAUfKttLearuWrP9MDH5MBPbIqV92AaeXatLxBI9gBaebbnrfifHhDYfgasaacH8akY=wiFfYdH8Gipec8Eeeu0xXdbba9frFj0=OqFfea0dXdd9vqai=hGuQ8kuc9pgc9s8qqaq=dirpe0xb9q8qiLsFr0=vr0=vr0dc8meaabaqaciaacaGaaeqabaqabeGadaaakeaaiiGacqWFdpWCdaqhaaWcbaGae8xSdeMae8NSdigabaGaeGOmaidaaaaa@32CB@, and σe2
 MathType@MTEF@5@5@+=feaafiart1ev1aaatCvAUfKttLearuWrP9MDH5MBPbIqV92AaeXatLxBI9gBaebbnrfifHhDYfgasaacH8akY=wiFfYdH8Gipec8Eeeu0xXdbba9frFj0=OqFfea0dXdd9vqai=hGuQ8kuc9pgc9s8qqaq=dirpe0xb9q8qiLsFr0=vr0=vr0dc8meaabaqaciaacaGaaeqabaqabeGadaaakeaaiiGacqWFdpWCdaqhaaWcbaGaemyzaugabaGaeGOmaidaaaaa@30E8@ are shown in Table 3.

To address the second aim (e.g., whether baseline PINP or ICTP predicts the change in BMD), the coefficients of time effects in model (1) was modeled as a function of baseline PINP or ICTP as follows: *β*_*i *_= *β *+ *ηPINP*_*i*0 _+ *s*_*i *_or *β*_*i *_= *β *+ *ηICTP*_*i*0 _+ *s*_*i *_Therefore the model becomes:

*BMD*_*it *_= *α *+ (*β *+ *ηPINP*_*i*0 _+ *s*_*i*_)*T*_*it *_+ *r*_*i *_+ *e*_*it*_

*BMD*_*it *_= *α *+ (*β *+ *ηICTP*_*i*0 _+ *s*_*i*_)*T*_*it *_+ *r*_*i *_+ *e*_*it *_    (4)

The estimates of *α*, *β*, *η*, σα2
 MathType@MTEF@5@5@+=feaafiart1ev1aaatCvAUfKttLearuWrP9MDH5MBPbIqV92AaeXatLxBI9gBaebbnrfifHhDYfgasaacH8akY=wiFfYdH8Gipec8Eeeu0xXdbba9frFj0=OqFfea0dXdd9vqai=hGuQ8kuc9pgc9s8qqaq=dirpe0xb9q8qiLsFr0=vr0=vr0dc8meaabaqaciaacaGaaeqabaqabeGadaaakeaaiiGacqWFdpWCdaqhaaWcbaGae8xSdegabaGaeGOmaidaaaaa@312F@, σβ2
 MathType@MTEF@5@5@+=feaafiart1ev1aaatCvAUfKttLearuWrP9MDH5MBPbIqV92AaeXatLxBI9gBaebbnrfifHhDYfgasaacH8akY=wiFfYdH8Gipec8Eeeu0xXdbba9frFj0=OqFfea0dXdd9vqai=hGuQ8kuc9pgc9s8qqaq=dirpe0xb9q8qiLsFr0=vr0=vr0dc8meaabaqaciaacaGaaeqabaqabeGadaaakeaaiiGacqWFdpWCdaqhaaWcbaGae8NSdigabaGaeGOmaidaaaaa@3131@, σαβ2
 MathType@MTEF@5@5@+=feaafiart1ev1aaatCvAUfKttLearuWrP9MDH5MBPbIqV92AaeXatLxBI9gBaebbnrfifHhDYfgasaacH8akY=wiFfYdH8Gipec8Eeeu0xXdbba9frFj0=OqFfea0dXdd9vqai=hGuQ8kuc9pgc9s8qqaq=dirpe0xb9q8qiLsFr0=vr0=vr0dc8meaabaqaciaacaGaaeqabaqabeGadaaakeaaiiGacqWFdpWCdaqhaaWcbaGae8xSdeMae8NSdigabaGaeGOmaidaaaaa@32CB@, and σe2
 MathType@MTEF@5@5@+=feaafiart1ev1aaatCvAUfKttLearuWrP9MDH5MBPbIqV92AaeXatLxBI9gBaebbnrfifHhDYfgasaacH8akY=wiFfYdH8Gipec8Eeeu0xXdbba9frFj0=OqFfea0dXdd9vqai=hGuQ8kuc9pgc9s8qqaq=dirpe0xb9q8qiLsFr0=vr0=vr0dc8meaabaqaciaacaGaaeqabaqabeGadaaakeaaiiGacqWFdpWCdaqhaaWcbaGaemyzaugabaGaeGOmaidaaaaa@30E8@ are shown in Table 4.

## Pre-publication history

The pre-publication history for this paper can be accessed here:



## Figures and Tables

**Table 1:** Characteristics of study subjects at baseline

**Variable**	**Mean (standard deviation)**	**5^th ^and 95^th ^quantile**
Age (yr)	70.4 (4.1)	66, 82
Weight (kg)	80.5 (12.3)	63, 102
Height (cm)	173.7 (6.4)	162, 183
Body mass index (kg/m^2^)	26.6 (3.4)	22.3, 34.3
sPINP(μg/L)	39.8 (13.1)	22.8, 67.1
sICTP (μg/L)	4.2 (1.1)	2.9, 6.8
Lumbar spine BMD (g/cm^2^)	1.29 (0.21)	0.98, 1.68
Femoral neck BMD (g/cm^2^)	0.95 (0.13)	0.73, 1.15
Duration of follow-up (yrs)	5.9 (1.5)	4.0, 8.9

sICTP, serum C-terminal telopeptide of type I collagen; sPINP, serum N-terminal propeptide of type I collagen; BMD bone mineral density.

**Table 2:** Rates of change in bone mineral density and bone turnover markers

	Symbol	Estimated parameters ± standard error (SE) for			
		
		Femoral neck BMD	Lumbar spine BMD	sPINP	sICTP
**Parameters**					
Baseline^a^	*α*	943.0 ± 13**	1288.0 ± 20.3**	40. 1 ± 1.3*	4.3 ± 0.12**
Rate of change^b^	*β*	-3.5 ± 1.0**	7.4 ± 1.3**	-0.4 ± 0.18*	0.08 ± 0.02*

**Variance components**					
Baseline	σα2 MathType@MTEF@5@5@+=feaafiart1ev1aaatCvAUfKttLearuWrP9MDH5MBPbIqV92AaeXatLxBI9gBaebbnrfifHhDYfgasaacH8akY=wiFfYdH8Gipec8Eeeu0xXdbba9frFj0=OqFfea0dXdd9vqai=hGuQ8kuc9pgc9s8qqaq=dirpe0xb9q8qiLsFr0=vr0=vr0dc8meaabaqaciaacaGaaeqabaqabeGadaaakeaaiiGacqWFdpWCdaqhaaWcbaGae8xSdegabaGaeGOmaidaaaaa@312F@	16650 ± 2453**	38968 ± 5883**	130.8 ± 25*	0.81 ± 0.22**
Rate of change	σβ2 MathType@MTEF@5@5@+=feaafiart1ev1aaatCvAUfKttLearuWrP9MDH5MBPbIqV92AaeXatLxBI9gBaebbnrfifHhDYfgasaacH8akY=wiFfYdH8Gipec8Eeeu0xXdbba9frFj0=OqFfea0dXdd9vqai=hGuQ8kuc9pgc9s8qqaq=dirpe0xb9q8qiLsFr0=vr0=vr0dc8meaabaqaciaacaGaaeqabaqabeGadaaakeaaiiGacqWFdpWCdaqhaaWcbaGae8NSdigabaGaeGOmaidaaaaa@3131@	44 ± 6*	2 ± 30	0.4 ± 0.51	0.01 ± 0.05
Covariance	σαβ2 MathType@MTEF@5@5@+=feaafiart1ev1aaatCvAUfKttLearuWrP9MDH5MBPbIqV92AaeXatLxBI9gBaebbnrfifHhDYfgasaacH8akY=wiFfYdH8Gipec8Eeeu0xXdbba9frFj0=OqFfea0dXdd9vqai=hGuQ8kuc9pgc9s8qqaq=dirpe0xb9q8qiLsFr0=vr0=vr0dc8meaabaqaciaacaGaaeqabaqabeGadaaakeaaiiGacqWFdpWCdaqhaaWcbaGae8xSdeMae8NSdigabaGaeGOmaidaaaaa@32CB@	43 ± 135	876 ± 283**	-1.1 ± 2.7	0.07 ± 0.03**
Random error	σe2 MathType@MTEF@5@5@+=feaafiart1ev1aaatCvAUfKttLearuWrP9MDH5MBPbIqV92AaeXatLxBI9gBaebbnrfifHhDYfgasaacH8akY=wiFfYdH8Gipec8Eeeu0xXdbba9frFj0=OqFfea0dXdd9vqai=hGuQ8kuc9pgc9s8qqaq=dirpe0xb9q8qiLsFr0=vr0=vr0dc8meaabaqaciaacaGaaeqabaqabeGadaaakeaaiiGacqWFdpWCdaqhaaWcbaGaemyzaugabaGaeGOmaidaaaaa@30E8@	908 ± 131**	3234 ± 327**	52.6 ± 6.8**	0.84 ± 0.08**

sICTP, serum C-terminal telopeptide of type I collagen; sPINP, serum N-terminal propeptide of type I collagen; BMD bone mineral density.

**Units of measurement**: ^a^baseline parameters were expressed in mg/cm^2 ^(for bone mineral density) and μg/L (for sPINP and sICTP); ^b^rate of change: mg/cm^2 ^per year (for bone mineral density) and μg/L per year (for sPINP and sICTP).

**Note of interpretation**: For femoral neck BMD, the overall average baseline measurement was 943 mg/cm^2^, and the rate of change was -3.5 mg/cm^2 ^per year. The standard deviation (SD) of baseline BMD among individuals was 16650
 MathType@MTEF@5@5@+=feaafiart1ev1aaatCvAUfKttLearuWrP9MDH5MBPbIqV92AaeXatLxBI9gBaebbnrfifHhDYfgasaacH8akY=wiFfYdH8Gipec8Eeeu0xXdbba9frFj0=OqFfea0dXdd9vqai=hGuQ8kuc9pgc9s8qqaq=dirpe0xb9q8qiLsFr0=vr0=vr0dc8meaabaqaciaacaGaaeqabaqabeGadaaakeaadaGcaaqaaiabigdaXiabiAda2iabiAda2iabiwda1iabicdaWaWcbeaaaaa@3191@ = 129 mg/cm^2^, and the SD of rates of change among individuals was 43
 MathType@MTEF@5@5@+=feaafiart1ev1aaatCvAUfKttLearuWrP9MDH5MBPbIqV92AaeXatLxBI9gBaebbnrfifHhDYfgasaacH8akY=wiFfYdH8Gipec8Eeeu0xXdbba9frFj0=OqFfea0dXdd9vqai=hGuQ8kuc9pgc9s8qqaq=dirpe0xb9q8qiLsFr0=vr0=vr0dc8meaabaqaciaacaGaaeqabaqabeGadaaakeaadaGcaaqaaiabisda0iabiodaZaWcbeaaaaa@2EB1@ = 6.6 mg/cm^2^. More importantly, the variance in BMD within individuals was 908 mg^2^/cm^4^. The covariance was not statistically significant, which suggests that there was no significant correlation between the baseline measurements and rates of change in BMD.

Statistical significance is indicated by *p < 0.05; ** 0.001 < p < 0.05, and *** p < 0.0001.

**Table 3:** Within-subject association between femoral neck BMD and bone turnover markers.

	**Symbol**	**Estimate ± SE**	**P-value**
**Analysis marker: sPINP**			
**Estimated parameters**			
Intercept (baseline)	*α*	944 ± 13	<0.0001
Rate of change	*β*	-3.7 ± 1.0	0.0004
sPINP	*γ*	-9.2 ± 4.4	0.022
**Variance components**			
Baseline	σα2 MathType@MTEF@5@5@+=feaafiart1ev1aaatCvAUfKttLearuWrP9MDH5MBPbIqV92AaeXatLxBI9gBaebbnrfifHhDYfgasaacH8akY=wiFfYdH8Gipec8Eeeu0xXdbba9frFj0=OqFfea0dXdd9vqai=hGuQ8kuc9pgc9s8qqaq=dirpe0xb9q8qiLsFr0=vr0=vr0dc8meaabaqaciaacaGaaeqabaqabeGadaaakeaaiiGacqWFdpWCdaqhaaWcbaGae8xSdegabaGaeGOmaidaaaaa@312F@	16428 ± 2425	<0.001
Rate of change	σβ2 MathType@MTEF@5@5@+=feaafiart1ev1aaatCvAUfKttLearuWrP9MDH5MBPbIqV92AaeXatLxBI9gBaebbnrfifHhDYfgasaacH8akY=wiFfYdH8Gipec8Eeeu0xXdbba9frFj0=OqFfea0dXdd9vqai=hGuQ8kuc9pgc9s8qqaq=dirpe0xb9q8qiLsFr0=vr0=vr0dc8meaabaqaciaacaGaaeqabaqabeGadaaakeaaiiGacqWFdpWCdaqhaaWcbaGae8NSdigabaGaeGOmaidaaaaa@3131@	38 ± 16	0.009
Covariance	σαβ2 MathType@MTEF@5@5@+=feaafiart1ev1aaatCvAUfKttLearuWrP9MDH5MBPbIqV92AaeXatLxBI9gBaebbnrfifHhDYfgasaacH8akY=wiFfYdH8Gipec8Eeeu0xXdbba9frFj0=OqFfea0dXdd9vqai=hGuQ8kuc9pgc9s8qqaq=dirpe0xb9q8qiLsFr0=vr0=vr0dc8meaabaqaciaacaGaaeqabaqabeGadaaakeaaiiGacqWFdpWCdaqhaaWcbaGae8xSdeMae8NSdigabaGaeGOmaidaaaaa@32CB@	72 ± 131	0.586
Random error	σe2 MathType@MTEF@5@5@+=feaafiart1ev1aaatCvAUfKttLearuWrP9MDH5MBPbIqV92AaeXatLxBI9gBaebbnrfifHhDYfgasaacH8akY=wiFfYdH8Gipec8Eeeu0xXdbba9frFj0=OqFfea0dXdd9vqai=hGuQ8kuc9pgc9s8qqaq=dirpe0xb9q8qiLsFr0=vr0=vr0dc8meaabaqaciaacaGaaeqabaqabeGadaaakeaaiiGacqWFdpWCdaqhaaWcbaGaemyzaugabaGaeGOmaidaaaaa@30E8@	903 ± 134	<0.001

**Analysis marker: sICTP**			
**Estimated parameters**			
Intercept (baseline)	*α*	943 ± 13	<0.0001
Rate of change	*β*	-3.4 ± 1.0	0.0013
sICTP	*γ*	-0.7 ± 3.6	0.8425
**Variance components**			
Baseline	σα2 MathType@MTEF@5@5@+=feaafiart1ev1aaatCvAUfKttLearuWrP9MDH5MBPbIqV92AaeXatLxBI9gBaebbnrfifHhDYfgasaacH8akY=wiFfYdH8Gipec8Eeeu0xXdbba9frFj0=OqFfea0dXdd9vqai=hGuQ8kuc9pgc9s8qqaq=dirpe0xb9q8qiLsFr0=vr0=vr0dc8meaabaqaciaacaGaaeqabaqabeGadaaakeaaiiGacqWFdpWCdaqhaaWcbaGae8xSdegabaGaeGOmaidaaaaa@312F@	16677 ± 2462	<0.0001
Rate of change	σβ2 MathType@MTEF@5@5@+=feaafiart1ev1aaatCvAUfKttLearuWrP9MDH5MBPbIqV92AaeXatLxBI9gBaebbnrfifHhDYfgasaacH8akY=wiFfYdH8Gipec8Eeeu0xXdbba9frFj0=OqFfea0dXdd9vqai=hGuQ8kuc9pgc9s8qqaq=dirpe0xb9q8qiLsFr0=vr0=vr0dc8meaabaqaciaacaGaaeqabaqabeGadaaakeaaiiGacqWFdpWCdaqhaaWcbaGae8NSdigabaGaeGOmaidaaaaa@3131@	44 ± 16	0.0041
Covariance	σαβ2 MathType@MTEF@5@5@+=feaafiart1ev1aaatCvAUfKttLearuWrP9MDH5MBPbIqV92AaeXatLxBI9gBaebbnrfifHhDYfgasaacH8akY=wiFfYdH8Gipec8Eeeu0xXdbba9frFj0=OqFfea0dXdd9vqai=hGuQ8kuc9pgc9s8qqaq=dirpe0xb9q8qiLsFr0=vr0=vr0dc8meaabaqaciaacaGaaeqabaqabeGadaaakeaaiiGacqWFdpWCdaqhaaWcbaGae8xSdeMae8NSdigabaGaeGOmaidaaaaa@32CB@	43 ± 135	0.7526
Random error	σe2 MathType@MTEF@5@5@+=feaafiart1ev1aaatCvAUfKttLearuWrP9MDH5MBPbIqV92AaeXatLxBI9gBaebbnrfifHhDYfgasaacH8akY=wiFfYdH8Gipec8Eeeu0xXdbba9frFj0=OqFfea0dXdd9vqai=hGuQ8kuc9pgc9s8qqaq=dirpe0xb9q8qiLsFr0=vr0=vr0dc8meaabaqaciaacaGaaeqabaqabeGadaaakeaaiiGacqWFdpWCdaqhaaWcbaGaemyzaugabaGaeGOmaidaaaaa@30E8@	907 ± 130	<0.0001

sICTP, serum C-terminal telopeptide of type I collagen; sPINP, serum N-terminal propeptide of type I collagen; BMD bone mineral density.

**Note of interpretation**: Results in part (a) suggest that the BMD measurement (mg/cm^2^) for a subject at time *t *(denoted by BMD_*t*_) can be represented by the equation: BMD_*t *_= 944 – 3.7*Year – 9.2* sPINP_*t*_, where "Year" is 0, 1, 2, ..., and sPINP_*t *_is the measurement of sPINP at time *t *for the subject. In other words, femoral neck BMD was decreased by -3.7 mg/cm^2 ^per year. Furthermore, each 1 μg/L increase in sPINP was associated with a 9.2 mg/cm^2 ^decrease in BMD. The interpretation of variance components is similar to that in Table 2. For example, the SD of baseline BMD among individuals was 16677
 MathType@MTEF@5@5@+=feaafiart1ev1aaatCvAUfKttLearuWrP9MDH5MBPbIqV92AaeXatLxBI9gBaebbnrfifHhDYfgasaacH8akY=wiFfYdH8Gipec8Eeeu0xXdbba9frFj0=OqFfea0dXdd9vqai=hGuQ8kuc9pgc9s8qqaq=dirpe0xb9q8qiLsFr0=vr0=vr0dc8meaabaqaciaacaGaaeqabaqabeGadaaakeaadaGcaaqaaiabigdaXiabiAda2iabiAda2iabiEda3iabiEda3aWcbeaaaaa@31A3@ = 129 mg/cm^2^, and the SD of rates of change among individuals was 44
 MathType@MTEF@5@5@+=feaafiart1ev1aaatCvAUfKttLearuWrP9MDH5MBPbIqV92AaeXatLxBI9gBaebbnrfifHhDYfgasaacH8akY=wiFfYdH8Gipec8Eeeu0xXdbba9frFj0=OqFfea0dXdd9vqai=hGuQ8kuc9pgc9s8qqaq=dirpe0xb9q8qiLsFr0=vr0=vr0dc8meaabaqaciaacaGaaeqabaqabeGadaaakeaadaGcaaqaaiabisda0iabisda0aWcbeaaaaa@2EB3@ = 6.6 mg/cm^2^. The within-individuals variance in BMD was 907 mg^2^/cm^4^, a slight reduction from 908 mg^2^/cm^4 ^(Table 2), which suggests that including sPINP did not have a substantial effect on the within-subjects variance in BMD.

**Table 4:** Association between baseline PINP, ICTP and change in femoral neck BMD

	**Symbol**	**Estimate ± SE**	**P-value**
**Analysis marker: sPINP**			
**Estimated parameters**			
Intercept (baseline)	*α*	943 ± 13	<0.0001
Rate of change	*β*	-3.5 ± 1.0	0.0004
sPINP	*γ*	0.7 ± 1.1	0.4953
**Variance components**			
Baseline	σα2 MathType@MTEF@5@5@+=feaafiart1ev1aaatCvAUfKttLearuWrP9MDH5MBPbIqV92AaeXatLxBI9gBaebbnrfifHhDYfgasaacH8akY=wiFfYdH8Gipec8Eeeu0xXdbba9frFj0=OqFfea0dXdd9vqai=hGuQ8kuc9pgc9s8qqaq=dirpe0xb9q8qiLsFr0=vr0=vr0dc8meaabaqaciaacaGaaeqabaqabeGadaaakeaaiiGacqWFdpWCdaqhaaWcbaGae8xSdegabaGaeGOmaidaaaaa@312F@	16661 ± 2454	<0.001
Rate of change	σβ2 MathType@MTEF@5@5@+=feaafiart1ev1aaatCvAUfKttLearuWrP9MDH5MBPbIqV92AaeXatLxBI9gBaebbnrfifHhDYfgasaacH8akY=wiFfYdH8Gipec8Eeeu0xXdbba9frFj0=OqFfea0dXdd9vqai=hGuQ8kuc9pgc9s8qqaq=dirpe0xb9q8qiLsFr0=vr0=vr0dc8meaabaqaciaacaGaaeqabaqabeGadaaakeaaiiGacqWFdpWCdaqhaaWcbaGae8NSdigabaGaeGOmaidaaaaa@3131@	44 ± 16	0.004
Covariance	σαβ2 MathType@MTEF@5@5@+=feaafiart1ev1aaatCvAUfKttLearuWrP9MDH5MBPbIqV92AaeXatLxBI9gBaebbnrfifHhDYfgasaacH8akY=wiFfYdH8Gipec8Eeeu0xXdbba9frFj0=OqFfea0dXdd9vqai=hGuQ8kuc9pgc9s8qqaq=dirpe0xb9q8qiLsFr0=vr0=vr0dc8meaabaqaciaacaGaaeqabaqabeGadaaakeaaiiGacqWFdpWCdaqhaaWcbaGae8xSdeMae8NSdigabaGaeGOmaidaaaaa@32CB@	54 ± 131	0.692
Random error	σe2 MathType@MTEF@5@5@+=feaafiart1ev1aaatCvAUfKttLearuWrP9MDH5MBPbIqV92AaeXatLxBI9gBaebbnrfifHhDYfgasaacH8akY=wiFfYdH8Gipec8Eeeu0xXdbba9frFj0=OqFfea0dXdd9vqai=hGuQ8kuc9pgc9s8qqaq=dirpe0xb9q8qiLsFr0=vr0=vr0dc8meaabaqaciaacaGaaeqabaqabeGadaaakeaaiiGacqWFdpWCdaqhaaWcbaGaemyzaugabaGaeGOmaidaaaaa@30E8@	902 ± 130	<0.001

**Analysis marker: sICTP**			
**Estimated parameters**			
Intercept (baseline)	*α*	943 ± 13	<0.0001
Rate of change	*β*	-3.6 ± 1.0	0.0008
sICTP	*γ*	-0.6 ± 1.3	0.629
**Variance components**			
Baseline	σα2 MathType@MTEF@5@5@+=feaafiart1ev1aaatCvAUfKttLearuWrP9MDH5MBPbIqV92AaeXatLxBI9gBaebbnrfifHhDYfgasaacH8akY=wiFfYdH8Gipec8Eeeu0xXdbba9frFj0=OqFfea0dXdd9vqai=hGuQ8kuc9pgc9s8qqaq=dirpe0xb9q8qiLsFr0=vr0=vr0dc8meaabaqaciaacaGaaeqabaqabeGadaaakeaaiiGacqWFdpWCdaqhaaWcbaGae8xSdegabaGaeGOmaidaaaaa@312F@	16643 ± 2453	<0.0001
Rate of change	σβ2 MathType@MTEF@5@5@+=feaafiart1ev1aaatCvAUfKttLearuWrP9MDH5MBPbIqV92AaeXatLxBI9gBaebbnrfifHhDYfgasaacH8akY=wiFfYdH8Gipec8Eeeu0xXdbba9frFj0=OqFfea0dXdd9vqai=hGuQ8kuc9pgc9s8qqaq=dirpe0xb9q8qiLsFr0=vr0=vr0dc8meaabaqaciaacaGaaeqabaqabeGadaaakeaaiiGacqWFdpWCdaqhaaWcbaGae8NSdigabaGaeGOmaidaaaaa@3131@	43 ± 16	0.0041
Covariance	σαβ2 MathType@MTEF@5@5@+=feaafiart1ev1aaatCvAUfKttLearuWrP9MDH5MBPbIqV92AaeXatLxBI9gBaebbnrfifHhDYfgasaacH8akY=wiFfYdH8Gipec8Eeeu0xXdbba9frFj0=OqFfea0dXdd9vqai=hGuQ8kuc9pgc9s8qqaq=dirpe0xb9q8qiLsFr0=vr0=vr0dc8meaabaqaciaacaGaaeqabaqabeGadaaakeaaiiGacqWFdpWCdaqhaaWcbaGae8xSdeMae8NSdigabaGaeGOmaidaaaaa@32CB@	56 ± 139	0.6848
Random error	σe2 MathType@MTEF@5@5@+=feaafiart1ev1aaatCvAUfKttLearuWrP9MDH5MBPbIqV92AaeXatLxBI9gBaebbnrfifHhDYfgasaacH8akY=wiFfYdH8Gipec8Eeeu0xXdbba9frFj0=OqFfea0dXdd9vqai=hGuQ8kuc9pgc9s8qqaq=dirpe0xb9q8qiLsFr0=vr0=vr0dc8meaabaqaciaacaGaaeqabaqabeGadaaakeaaiiGacqWFdpWCdaqhaaWcbaGaemyzaugabaGaeGOmaidaaaaa@30E8@	907 ± 131	<0.0001

sICTP, serum C-terminal telopeptide of type I collagen; sPINP, serum N-terminal propeptide of type I collagen; BMD bone mineral density.

**Note of interpretation**: Results in part (a) suggest that the BMD measurement (mg/cm^2^) for a subject at time *t *(denoted by BMD_*t*_) can be represented by the equation: BMD_*t *_= 943 – 3.5*Year – 9.2* sPINP0, where "Year" is 0, 1, 2, ..., and sPINP0 is the measurement of sPINP at baseline for the subject. In other words, femoral neck BMD was decreased by -3.5 mg/cm^2 ^per year. There was no significant association between baseline sPINP and change in BMD (p = 0.4953). The interpretation of variance components is similar to that in Table 3.
